# Exploring the common pathogenesis of Alzheimer’s disease and type 2 diabetes mellitus *via* microarray data analysis

**DOI:** 10.3389/fnagi.2023.1071391

**Published:** 2023-02-27

**Authors:** Xian-wen Ye, Meng-nan Liu, Xuan Wang, Shui-qing Cheng, Chun-shuai Li, Yu-ying Bai, Lin-lin Yang, Xu-xing Wang, Jia Wen, Wen-juan Xu, Shu-yan Zhang, Xin-fang Xu, Xiang-ri Li

**Affiliations:** ^1^Centre of TCM Processing Research, Beijing University of Chinese Medicine, Beijing, China; ^2^Beijing Key Laboratory for Quality Evaluation of Chinese Materia Medica, Beijing University of Chinese Medicine, Beijing, China; ^3^School of Traditional Chinese Medicine, Beijing University of Chinese Medicine, Beijing, China

**Keywords:** network pharmacology, Alzheimer’s disease, type 2 diabetes mellitus, bioinformatics analysis, pathophysiological mechanisms

## Abstract

**Background:**

Alzheimer’s Disease (AD) and Type 2 Diabetes Mellitus (DM) have an increased incidence in modern society. Although more and more evidence has supported that DM is prone to AD, the interrelational mechanisms remain fully elucidated.

**Purpose:**

The primary purpose of this study is to explore the shared pathophysiological mechanisms of AD and DM.

**Methods:**

Download the expression matrix of AD and DM from the Gene Expression Omnibus (GEO) database with sequence numbers GSE97760 and GSE95849, respectively. The common differentially expressed genes (DEGs) were identified by limma package analysis. Then we analyzed the six kinds of module analysis: gene functional annotation, protein–protein interaction (PPI) network, potential drug screening, immune cell infiltration, hub genes identification and validation, and prediction of transcription factors (TFs).

**Results:**

The subsequent analyses included 339 common DEGs, and the importance of immunity, hormone, cytokines, neurotransmitters, and insulin in these diseases was underscored by functional analysis. In addition, serotonergic synapse, ovarian steroidogenesis, estrogen signaling pathway, and regulation of lipolysis are closely related to both. DEGs were input into the CMap database to screen small molecule compounds with the potential to reverse AD and DM pathological functions. L-690488, exemestane, and BMS-345541 ranked top three among the screened small molecule compounds. Finally, 10 essential hub genes were identified using cytoHubba, including *PTGS2, RAB10, LRRK2, SOS1, EEA1, NF1, RAB14, ADCY5, RAPGEF3,* and *PRKACG*. For the characteristic Aβ and Tau pathology of AD, *RAPGEF3* was associated significantly positively with AD and *NF1* significantly negatively with AD. In addition, we also found *ADCY5* and *NF1* significant correlations with DM phenotypes. Other datasets verified that *NF1*, *RAB14*, *ADCY5,* and *RAPGEF3* could be used as key markers of DM complicated with AD. Meanwhile, the immune cell infiltration score reflects the different cellular immune microenvironments of the two diseases.

**Conclusion:**

The common pathogenesis of AD and DM was revealed in our research. These common pathways and hub genes directions for further exploration of the pathogenesis or treatment of these two diseases.

## Introduction

Type 2 diabetes mellitus (DM) is a complex disease characterized by insulin resistance, the neurodegenerative mechanisms are inflammation, endoplasmic reticulum stress, autophagy, and mitochondrial dysfunction (Burillo et al., 2021). The primary pathology of Alzheimer’s disease (AD) is the accumulation of amyloid β (Aβ) and tau hyperphosphorylation. Insulin action and impaired glucose metabolism are also involved in the occurrence and development of AD. Pathological features similar to DM in the brains of patients with AD were observed, such as insulin efficacy and lack of glucose metabolism (Takeishi et al., 2021). A study used weighted gene co-expression network analysis to discover the common mechanisms of AD and DM, such as circadian entrainment, phagosomes, and glutathione metabolism (Zhu et al., 2020). These characteristics suggest that AD may be associated with DM, leading to a new term, type 3 diabetes (Diniz Pereira et al., 2021). Both DM and AD occur commonly in elderly people, and DM has been considered a potential critical risk factor for AD (Wang et al., 2017). DM increases the risk of dementia in carriers with the APOE ɛ4 allele, and the heritability of the two diseases is estimated to be more than 50% (Li et al., 2020). A meta-analysis of 28 observational studies shows that people with DM are more likely to develop AD. Compared with non-diabetic patients people with a history of diabetes had a 73% increase in the risk of all types of dementia, a 56% increase in AD, and a 127% increase in vascular dementia (Gudala et al., 2013). AD and DM share many pathophysiological characteristics, comprising defects in glucose transporters, mitochondrial dysfunctions in the brain, impaired insulin sensitivity, Aβ accumulation, tau hyperphosphorylation, brain vasculopathy, inflammation, and oxidative stress (Tumminia et al., 2018). For instance, the activation of glycogen synthase kinase 3β requires insulin, which in turn causes tau phosphorylation to form neuronal fiber tangles. Interestingly, not only insulin significantly contributes to the formation of amyloid plaques but also amylin co-secreted with insulin favors this process (Kandimalla et al., 2017). Chronic hyperglycemia also leads to neuroinflammation and tau hyperphosphorylation in the hippocampus leading to cognitive decline (Wirt et al., 2021). Studies have shown that Aβ deposition and tau phosphorylation might be achieved through altered insulin pathways, both leading factors for AD development (Boccardi et al., 2019). Neuroinflammation is a recognized central mechanism of aging-related diseases, such as cognitive impairment and diabetes. To further add to these injuries, adult neurogenesis that provides neuronal plasticity is also impaired in the diabetic brain (Pugazhenthi et al., 2017). It has been found that low-dose STZ-induced hyperglycemia impairs network activity in the hippocampus and anterior cingulate cortex, mainly by increasing the phosphorylation of tau in the hippocampus and cortex (Wirt et al., 2021). Studies have shown that a vanadium compound bis(ethyl maltol to)-oxovanadium (IV), used initially to treat DM, effectively improves the inflammation in the brain of AD mice, significantly reduces the level of Aβ, and the spatial learning and memory activities of AD mice revised substantially (He et al., 2021). Since DM is a long-term chronic disease, it takes some time to develop into AD, and more attention should be paid to protecting brain function to avoid AD during DM treatment (Li et al., 2021).

The common transcriptional feature provided a novel and feasible scheme for the common pathogenesis of AD and DM at the genetic level. We analyzed the two gene expression matrix (GSE97760 and GSE95849). Comprehensive bioinformatics and enrichment analysis will determine common DEGs and their function on AD and DM. In addition, the PPI network was constructed with the STRING database and analyzed with Cytoscape software. ImmuCellAI is a reliable and efficient platform for immune infiltration analysis, successfully quantifying 24 immune cell subsets of AD and DM. Finally, we identified and validated 10 significant representative hub genes in AD and DM. In addition, we validated the transcription factors of these genes and their expression in the final analysis. Revealing the hub genes of AD and DM helps clarify the common mechanism between them. It provides a new means for exploring the molecular biological mechanism of other multiple diseases.

## Materials and methods

### Data source

GEO,^1^ containing many high-throughput sequencing and microarray gene sets, is a public database submitted by research institutes worldwide (Edgar et al., 2002). We searched for related gene expression datasets using Alzheimer’s disease (AD) and Type 2 Diabetes mellitus (DM) as keywords. Two microarray datasets [GSE97760 and GSE95849] from the blood genome were downloaded from GEO(Agilent GPL16699 platform, Phalanx Human lncRNA One Array v1_mRNA GPL22448 platform). The GSE97760 dataset contains patients with AD (*n* = 9) and healthy controls (*n* = 10) (Naughton et al., 2015). GSE95849 consists of patients with DM (*n* = 6) and healthy female controls (*n* = 6) from the peripheral blood mononuclear cells (Luo et al., 2017).

### Identification of DEGs

The GEO query package reads matrix data in Rstudio. Remove probe sets without gene symbols and take their maximum values for genes with multiple probe sets. Only the genes value with *p* < 0.05 and |logFC| ≥ 1 were identified as DEGs. The Funrich was used to obtain the common DEGs between AD and DM.

### Enrichment analyses of DEGs

Gene ontology (GO) is a multifaceted annotation of the genome for biological processes, cellular components, and molecular functions (Ye et al., 2022). The Kyoto Encyclopedia of Genome and Genome (KEGG) annotates the genetic pathways of different species in many ways, providing information about biological functions (Ye et al., 2020). The GO and KEGG pathways were enriched by the omicshare database.^2^ The GO enrichment results were plotted by Chiplot.^3^ *p* < 0.05 was considered significant.

### Construction of protein–protein interaction networks and prediction of small molecule drugs

Search Tool for the Retrieval of Interacting Genes (STRING; http://string-db.org) (version 11.5) constructed an interaction network between genes with a combined score of over 0.4 (Deng et al., 2020b). Cytoscape^4^ (version 3.9.0) observes the connections between targets and can be used to visualize this PPI network (Deng et al., 2020a). Using the Connectivity Map (CMap, https://clue.io/) database, DEGs were compared with a reference dataset, pert type selected for perturbation types, and trt cp selected for compounds. A connectivity score was obtained according to the enrichment of DEGs in the reference gene expression profile. A negative correlation analysis was performed to predict small molecule drugs capable of reversing the pathology of the disease (Subramanian et al., 2017).

### Analysis of immune cell infiltration

Immune Cell Abundance Identifier (ImmuCellAI, http://bioinfo.life.hust.edu.cn/ImmuCellAI#!/), a widely used database for evaluating cell infiltration in the microenvironment (Healy et al., 2008). ImmuCellAI can predict the abundance of 24 immune cell types in samples. The immune cell infiltration in different groups will be analyzed with ImmuCellAI in the examined group. Using the ImmuCellA algorithm, the study analyzed patients with AD or DM data and quantified the relative proportion of 24 infiltrating immune cells.

### Selection and analysis of hub genes

This study used the cytoHubba plugin of Cytoscape to identify hub genes and nine standard algorithms (MCC, Stress, Betweenness, Closeness, MNC, DMNC, Degree, Radiality, EPC) to evaluate and select hub genes. Subsequently, based on these hub genes, we constructed a co-expression network *via* GeneMANIA,^5^ a reliable and efficient bioinformatics tool for mining the intrinsic links between genes through the multi-angle of literature data (Warde-Farley et al., 2010).

### Prediction and verification of transcription factors

iRegulon implements a genome-wide ranking and recovery approach to detect enriched transcription factor motifs and their optimal sets of direct targets (Janky et al., 2014). Subsequently, we verified the TFs that regulate the hub genes and the expression levels of these TFs in GSE97760 and GSE95849 with the *T*-test, a *p*-value <0.05 was considered significant.

## Results

### Identification of DEGs

**Figure 1:**
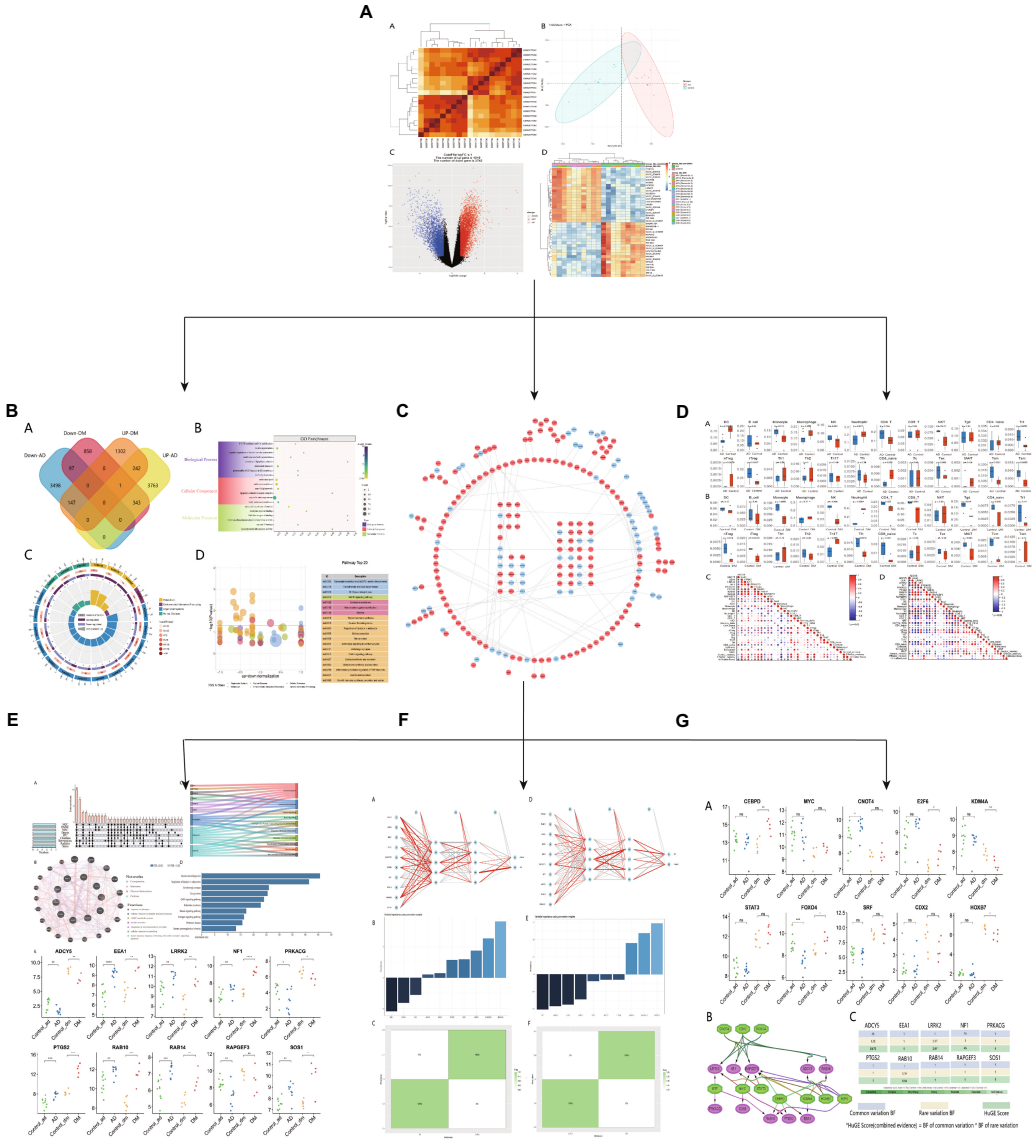
Research design flow chart. **(A)** Download AD and DM blood transcriptome data from GEO and analyze, **(B)** functional enrichment analysis of common differential genes, **(C)** PPI network map of common genes, **(D)** DM or AD immune infiltration analysis, **(E)** Hub gene expression level and co-expression network analysis, **(F)** Hub gene neural network analysis, **(G)** Hub gene TFs expression level and phenotypic correlation analysis.

**Figure 2:**
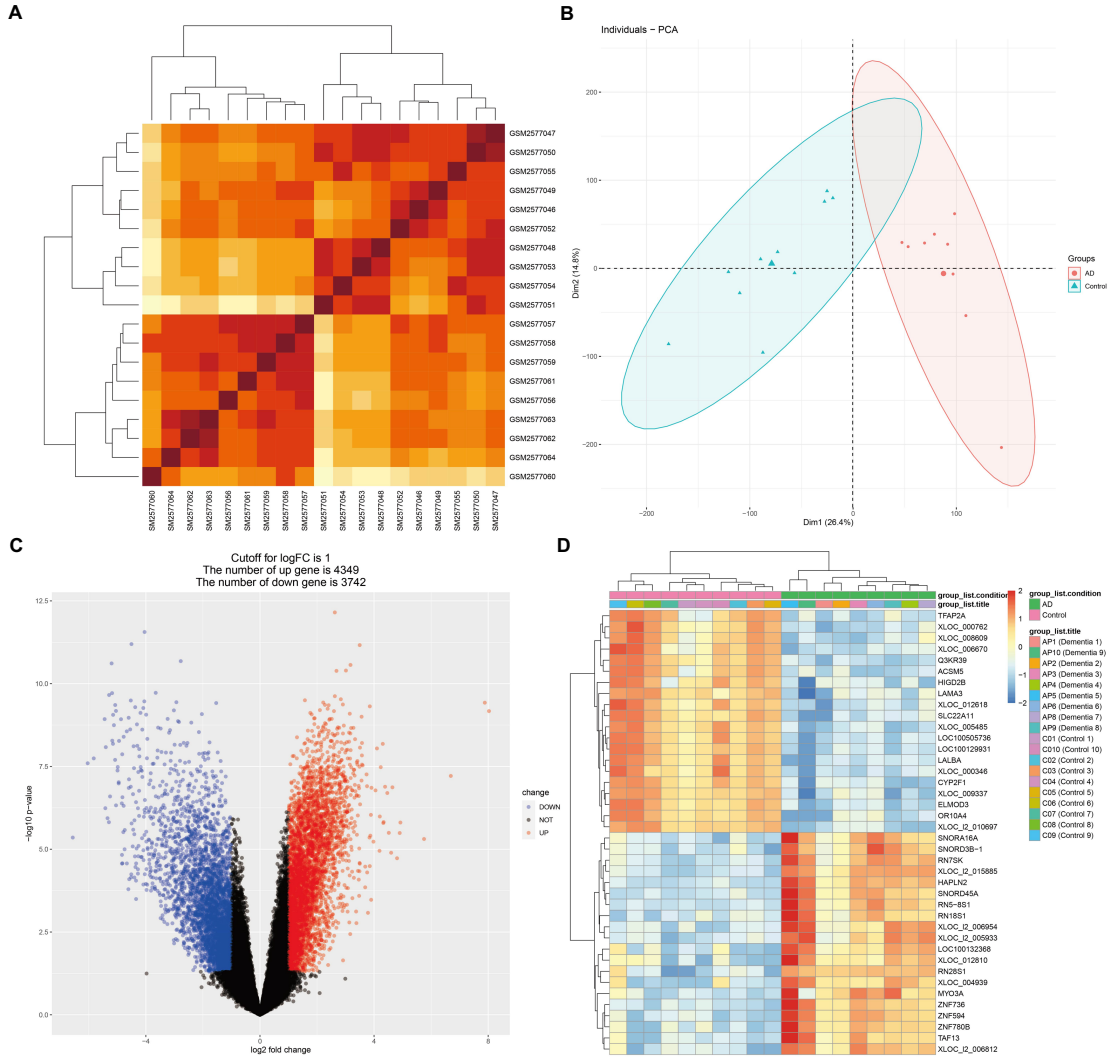
Microarray normalization and differential gene analysis in the AD group. **(A)** The heatmap of AD, **(B)** The PCA map of AD, **(C)** The volcano map of AD, and **(D)** Differential heatmap of partial gene expression between AD and normal group.

**Figure 3:**
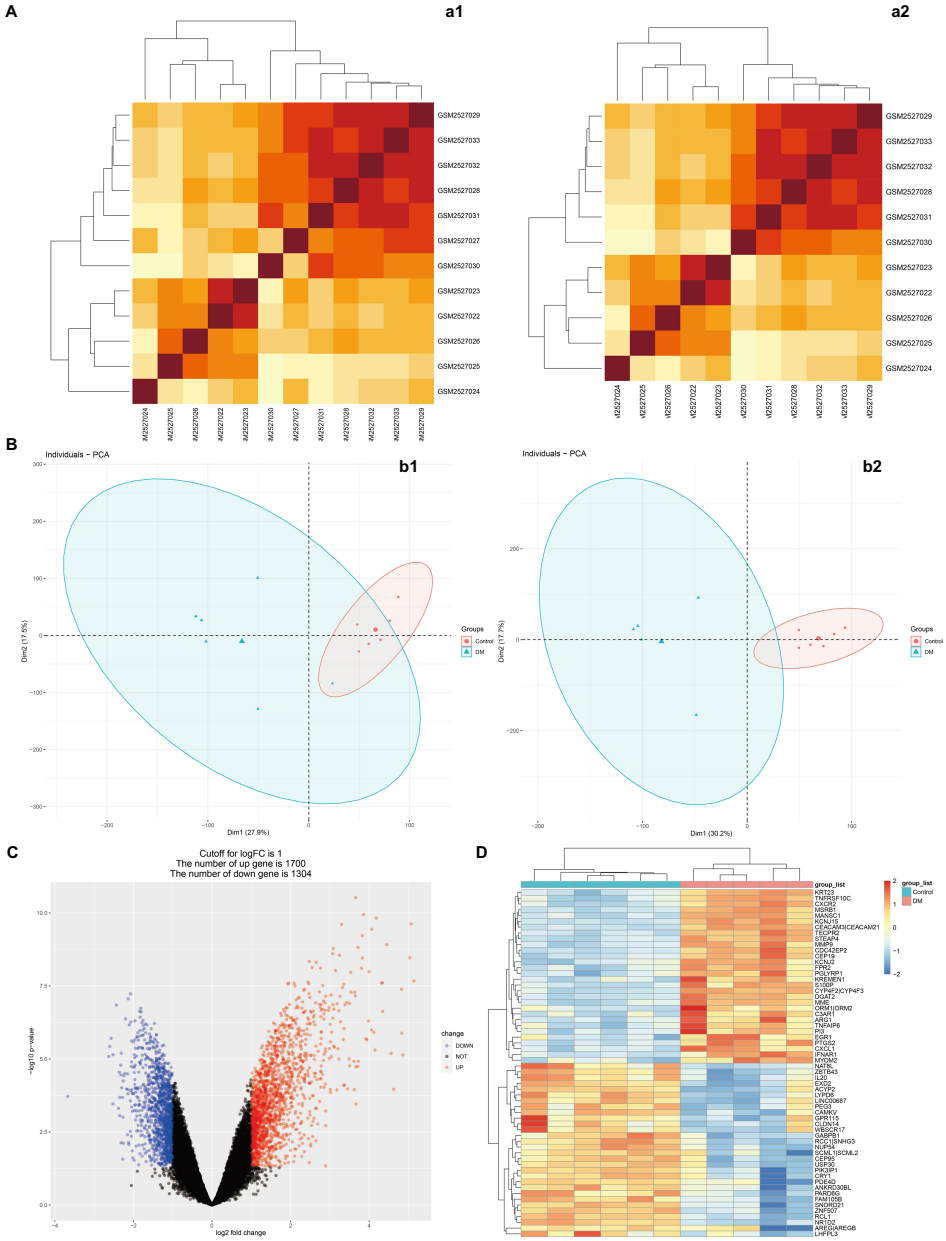
Microarray normalization and differential gene analysis in the DM group. **(A)** The heatmap of DM, **(B)** The PCA map of DM, **(C)** The volcano map of DM, and **(D)** Differential heatmap of partial gene expression between DM and normal group. The data is represented by a1/b1 before processing and a2/b2 after processing.

**Figure 4:**
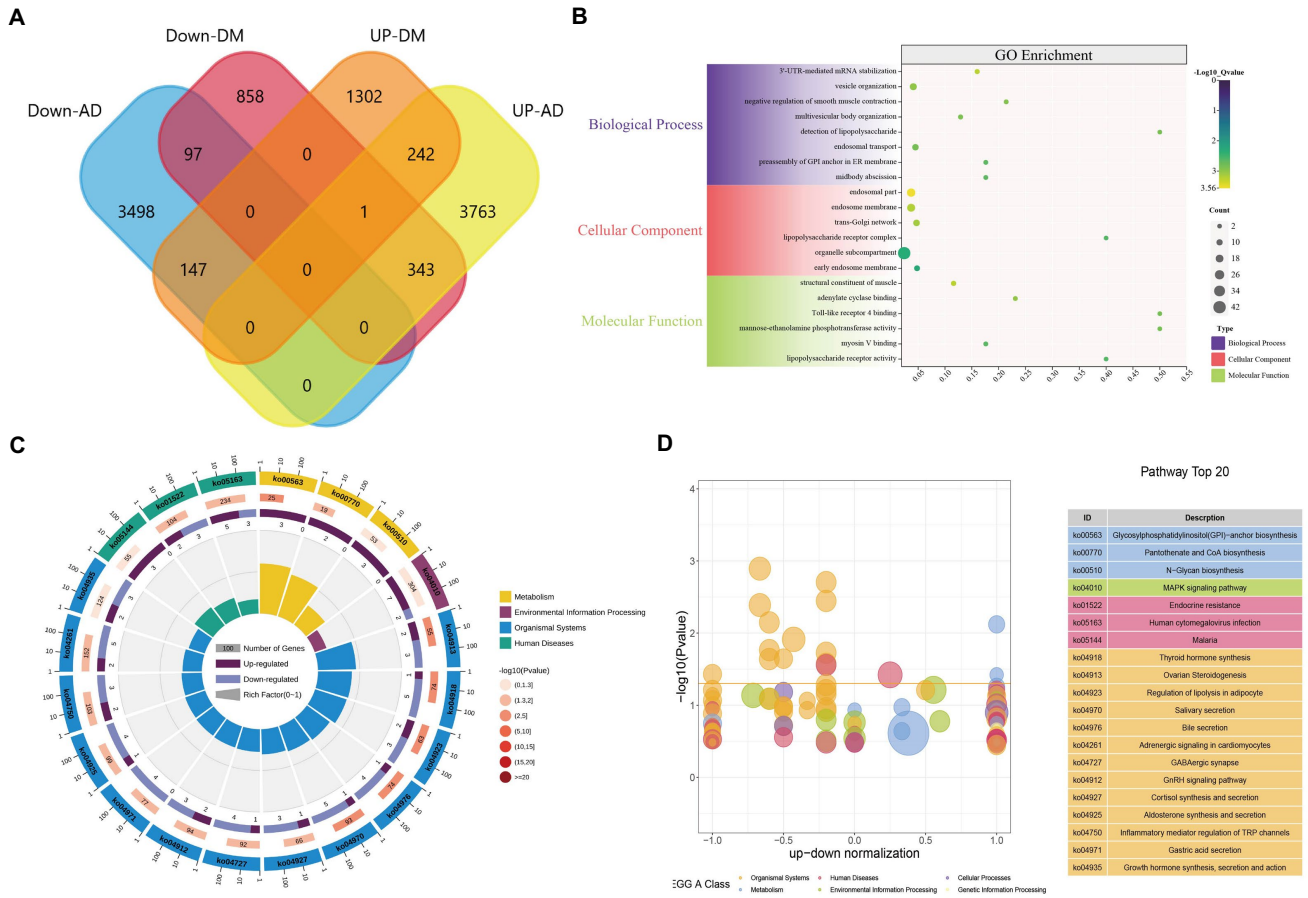
DEGs enrichment analysis results. **(A)** The two datasets showed an overlap of 339 DEGs, **(B)** The enrichment analysis results of GO, and **(C–D)** The enrichment analysis results of the KEGG Pathway. Adjusted *p*-value <0.05 was considered significant.

Figure 1 presents the idea of the article. After correlation analysis, standardizing processing of the microarray results (Figures 2A,B, 3A,B), DEGs (8,091 in GSE97760 and 3,004 in GSE95849) were identified (Figures 2C,D, 3C,D). The 339 common DEGs (97 down-regulated, 242 upregulated) were obtained after excluding genes with opposite expression trends in GSE97760 between GSE95849 (Figures 4A). In the DEGs analysis, GSM2527027 was treated as an outlier sample, so this sample was removed during the subsequent analysis (Figures 3A:a1,a2,B:b1,b2).

### Analysis of the functional features of common DEGs

GO functions and KEGG Pathway enrichment analyses were performed to analyze the biological functions and pathways involved in the 339 common DEGs (Supplementary Table S1). GO analysis results show that 4,236 biological process (BP), which contains 3’-UTR-mediated mRNA stabilization, vesicle organization, cytokinetic process, and astrocyte development; 567 cellular components (CC), involved endosomal part, lipopolysaccharide receptor complex, Wnt signalosome and glycosylphosphatidylinositol-mannosyltransferase I complex; 720 molecular functions (MF), such as structural constituent of muscle, Toll-like receptor 4 binding, lipopolysaccharide receptor activity and Toll-like receptor binding (Figure 4B). KEGG Pathway includes organismal systems, metabolism, environmental information processing, and human diseases. Three significant enrichment pathways in terms of metabolism are glycan biosynthesis and metabolism, metabolism of cofactors, glycan biosynthesis and metabolism; thyroid hormone synthesis, ovarian steroidogenesis, and regulation of lipolysis in adipocytes were enriched in organismal systems; MAPK signaling pathway, cAMP signaling pathway and Hippo signaling pathway found in environmental information processing; endocrine resistance, malaria, legionellosis classed in human diseases (Figures 4C,D). These results indicate that inflammatory, hormones, cytokines, and glycan are jointly involved in the occurrence and development of AD and DM.

### PPI network construction and small molecule drug prediction

**Figure 5:**
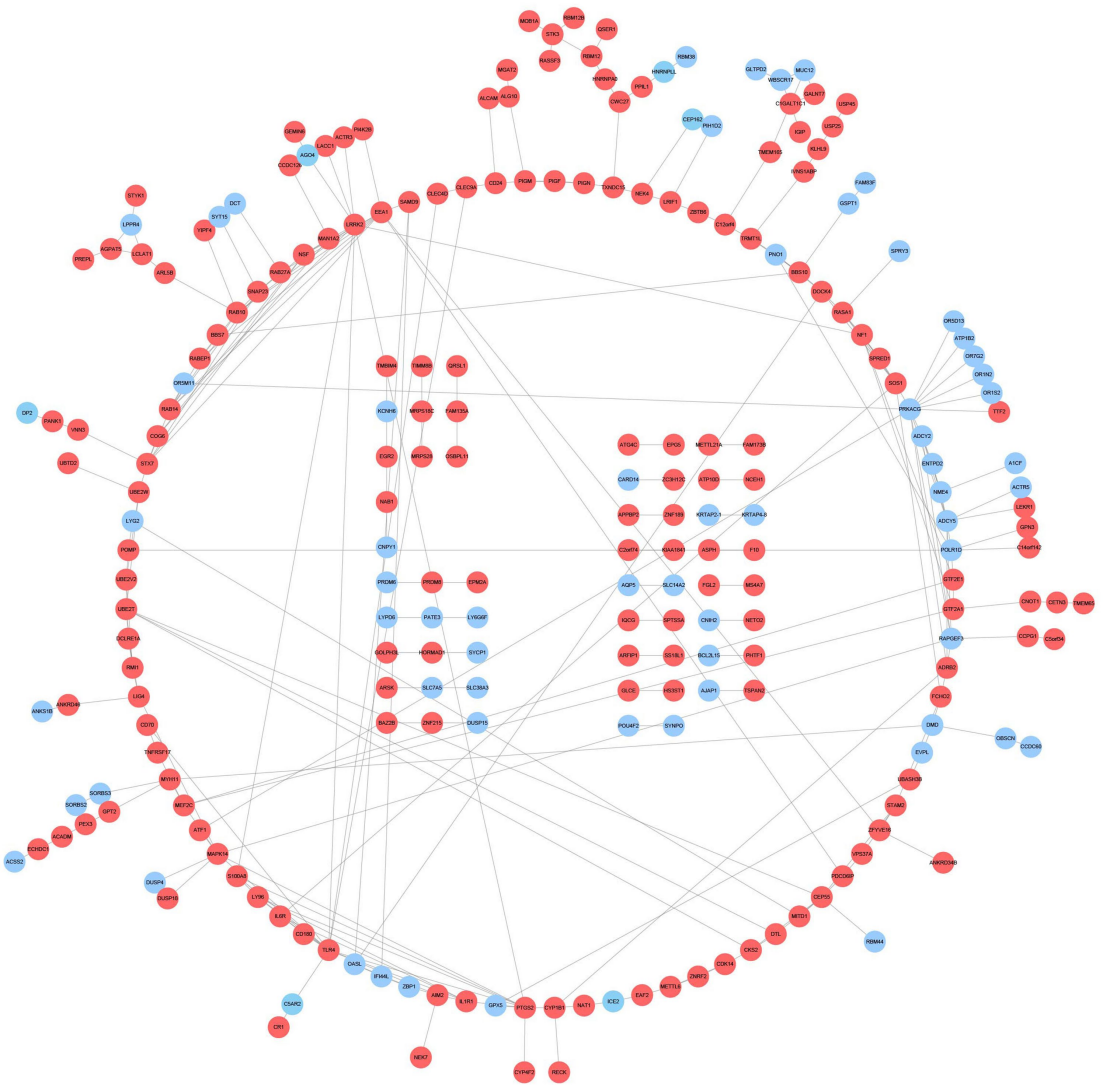
PPI network and common DEGs. Red indicates upregulated genes, and blue-violet indicates down-regulated genes.

The PPI network contains 233 nodes and 286 interaction pairs (Figure 5). DEGs were input into the CMap database to predict small molecule compounds that may reverse two diseases’ pathology by connectivity (Supplementary Table S2). To explore the feasibility of this method, we searched the approved drugs for both disorders in Drugbank^6^ and obtained 10 drugs for AD and 52 medications for DM (Supplementary Table S3). Interestingly, in the CMap results, seven drugs for AD were matched with a score range of −0.9247 to −1.3658; similarly, 27 pills for DM were obtained with a score range of −0.6906 to −1.0733. Among the 7,952 negatively correlated small molecule compounds, the score range of the top 10 small molecules is −1.6824 to −1.8921, which is significantly lower than the listed drugs of the two diseases, suggesting that these small molecules have the potential to reverse two diseases’ pathology.

### Immune infiltration analyses

**Figure 6:**
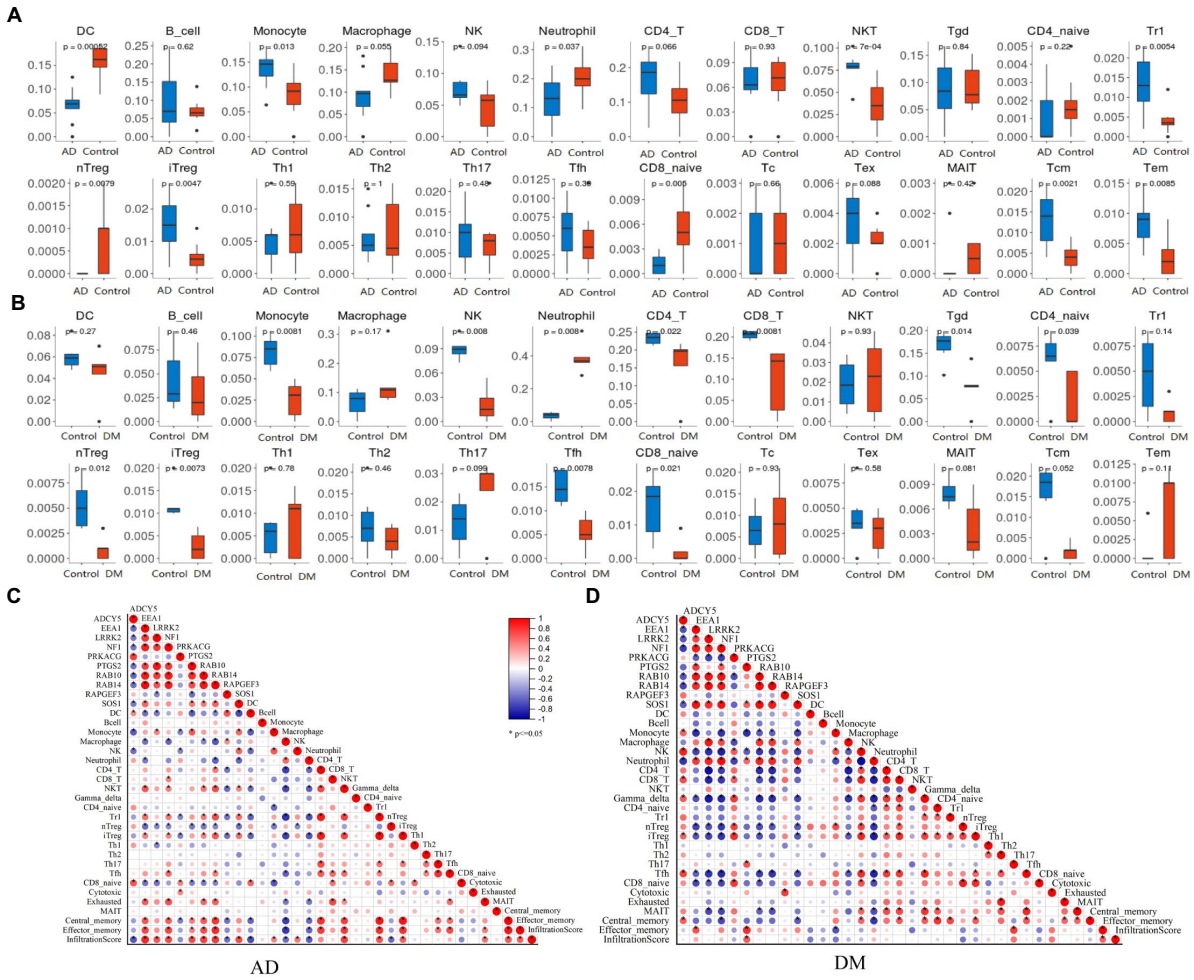
Immune infiltration analysis. **(A)** Analysis of immune infiltration in an AD group (blue) and blank group (red), **(B)** Analysis of immune infiltration in DM group (red) and blank group (blue), **(C)** Analysis of the relationship between Hub gene and immune infiltration in the AD group, and **(D)** Analysis of the relationship between Hub gene and immune infiltration in the DM group. Unpaired *t*-test, Mean ± SD, *p*-value <0.05 was considered significant.

Detecting the microenvironment has a significant reference value for clinical treatment sensitivity and disease diagnosis (Liu et al., 2021). After studying the relationship between immune infiltration and gene matrix, we further explored the potential molecular mechanism of genes affecting the progression of the two diseases (Figure 6). The results indicated that the AD group’s fractions for monocytes, NKT, Tr1, iTreg, Tcm, and Tem were remarkably higher than those of the regular patients. In comparison, the particles of many cells are lower than those of normal patients, such as DC, Neutrophil, nTreg, and CD8_navie (Figure 6A). However, the condition of immune infiltration behaved differently in the DM group. Compared with regular patients, Neutrophil increased significantly in the DM group, whereas monocytes, iTreg, and iTreg decreased significantly, and other significant decreases were NK, CD4_T, CD8_T, Tgd, CD4_navie, nTreg, Tfh, and CD8_naive (Figure 6B). The above results reflect the different cellular immune microenvironments of various diseases.

### Selection and analysis of hub genes

**Figure 7:**
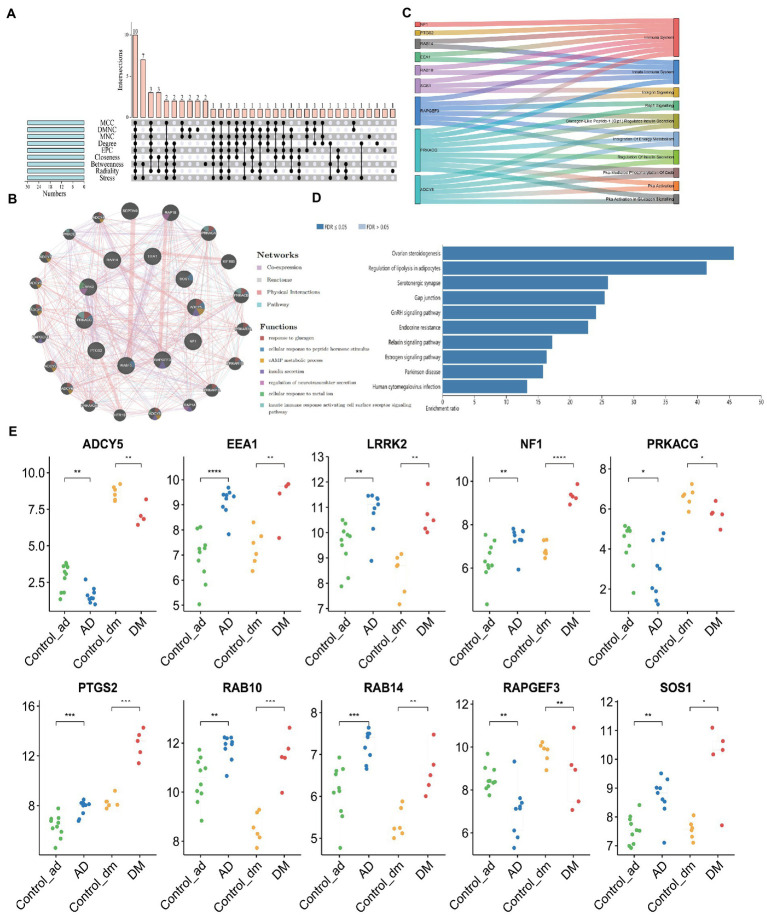
Acquisition of Hub Gene and Analysis of Co-expression Network. **(A)** The Venn diagram showed that nine algorithms have screened out 10 overlapping hub genes, **(B)** Ten hub genes and their co-expression network were analyzed by GeneMANIA, **(C)** Functional distribution of Hub gene, **(D)** Pathway analysis with WebGestalt, and **(E)** The mRNA expression of 10 Hub genes. Unpaired *t*-test, Mean ± SD, *p*-value <0.05 was considered significant, *p<0.05, **p<0.01, ***p<0.001.

According to the characteristics of the nine algorithms of cybHubba, we obtain the first 30 hub genes, respectively (Supplementary Table S4). Notably, these hub genes share 10 targets, including seven upregulated genes (*PTGS2*, *RAB10*, *LRRK2*, *SOS1*, *EEA1*, *NF1*, and *RAB14*) and three down-regulated genes (*ADCY5*, *RAPGEF3*, *PRKACG*) (Figure 7A). Table 1 shows the full name and the hub genes of related functions. Based on the GeneMANIA database, we got a complex PPI network with a co-expression of 59.87%, Reactome of 31.95%, physical interactions of 7.09%, and pathway of 1.10%. GO analysis involved response to glucagon, cellular response to peptide hormone stimulus, cAMP metabolic process, insulin secretion, regulation of neurotransmitter secretion, cellular response to the metal ion, and innate immune response activating cell surface receptor signaling pathway. These results of Reactome emphasized the critical role of the immune system and insulin in AD and DM (Figure 7B). Furthermore, pathway analysis with WebGestalt is associated with the serotonergic synapse, ovarian steroidogenesis, and estrogen signaling pathway, regulation of lipolysis in adipocytes, and human cytomegalovirus infection (Figure 7D). Interestingly, two genes (*ADCY5* and *PRKACG*) were almost involved in all top 10 KEGG Pathways (Figure 7C). Thus, neurotransmitters, insulin, immunity, and sex hormones play essential roles in developing these two diseases. Figure 7E shows the mRNA expression of 10 Hub genes.

**Table 1:** The details of the hub genes.

Gene Symbol	Gene name	Attribute	Functions
ADCY5	adenylate cyclase 5	Pka Activation, Pka-Mediated Phosphorylation of Creb, Glucagon-Like Peptide-1 (Glp1) Regulates Insulin Secretion, Regulation of Insulin Secretion, Pka Activation In Glucagon Signalling	activation of protein kinase activity
EEA1	early endosome antigen 1	Immune System, Innate Immune System	cytosolic transport
LRRK2	leucine-rich repeat kinase 2	cellular response	activation of protein kinase activity
NF1	neurofibromin 1	Immune System	negative regulation of kinase activity
PRKACG	protein kinase cAMP-activated catalytic subunit gamma	Pka-Mediated Phosphorylation of Creb, Glucagon-Like Peptide-1 (Glp1) Regulates Insulin Secretion, Pka Activation, Immune System, Integration of Energy Metabolism, Rap1 Signalling, Regulation of Insulin Secretion, Pka Activation In Glucagon Signalling, Innate Immune System	activation of innate immune response
PTGS2	prostaglandin-endoperoxide synthase 2	Immune System	nucleoside phosphate biosynthetic process
RAB10	RAB10, member RAS oncogene family	Immune System, Innate Immune System	cellular response to peptide
RAB14	RAB14, member RAS oncogene family	Immune System, Innate Immune System	cytosolic transport
RAPGEF3	Rap guanine nucleotide exchange factor 3	Immune System, Glucagon-Like Peptide-1 (Glp1) Regulates Insulin Secretion, Integration of Energy Metabolism, Integrin Signaling, Rap1 Signalling, Regulation of Insulin Secretion	cyclic-nucleotide-mediated signaling
SOS1	SOS Ras/Rac guanine nucleotide exchange factor 1	Immune System, Innate Immune System, Integrin Signaling	cellular response to peptide

To explore the contribution of the Hub gene to the immune infiltration of disease, we carried out a correlation analysis. For AD patients, *EEA1*, *LRRK2*, *NF1*, *PTGS2*, *RAB10*, *RAB14*, and *SOS1* were significantly positively associated with immune infiltration scores, and *ADCY5*, *RAPGEF3* were significantly negatively associated with immune infiltration scores (Figure 6C). And only *PTGS2* was significantly positively associated with immune infiltration scores in DM patients (Figure 6D).

**Figure 8:**
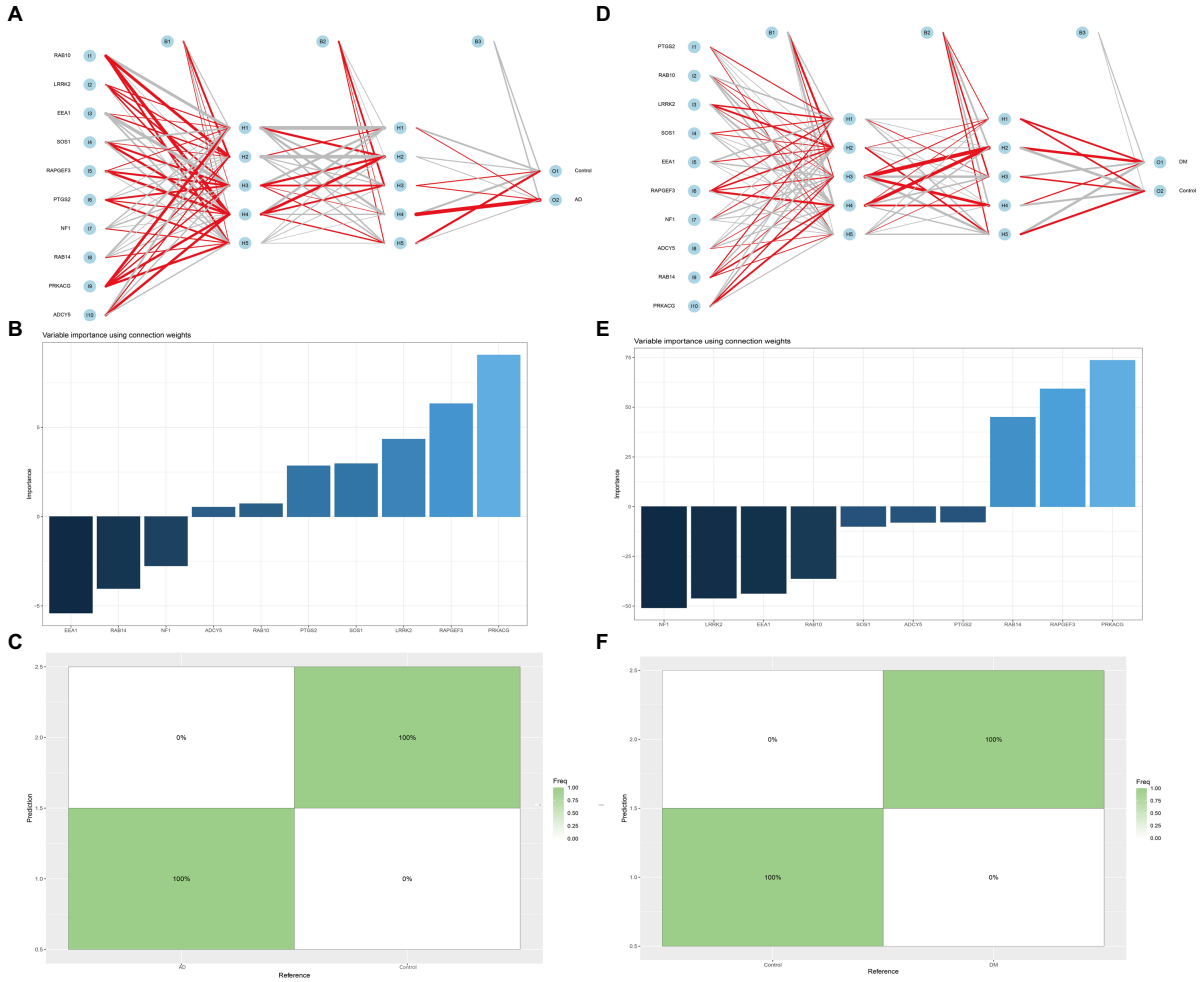
Multilayer perceptron networks analysis. **(A,D)** Neural network analysis of Hub gene in AD and DM groups, **(B,E)** The importance of each independent variable to the AD or DM model prediction, and **(C,F)** The prediction effect of the MLP classifier on the dataset of AD or DM.

In addition, we established multilayer perceptron (MLP) networks, which have two hidden layers and five neurons in each hidden layer. For the obtained mlpcla model, the network structure of the model can be visualized using the plotnet () function. After running the program, the image shown in Figure 8 can be obtained. In the connection weights between neurons in the network, the positive importance uses red lines, the negative consequence uses gray streaks, and the line’s thickness reflects the weight’s size (Figures 8A,D). For the MLP network, the importance of each independent variable to the model prediction results can be computed and visualized. Our study found that the top three most positive important variables for AD classification were *PRKACG*, *RAPGEF3*, and *LRRK2*. Three negative weights, such as *EEA1*, *RAB14*, and *NF1*. The top three most positive variables for DM classification were *PRKACG*, *RAPGEF3*, and *RAB14*.

Similarly, the negative weights are *NF1*, *LRRK2*, *EEA1*, etc. (Figures 8B,E). The prediction effect of the MLP classifier on the dataset can also be visualized using the confusion matrix. As shown in Figure 8, the seeds of AD and DM can be found in the confusion matrix, which can be predicted 100% correctly (Figures 8C,F).

**Figure 9:**
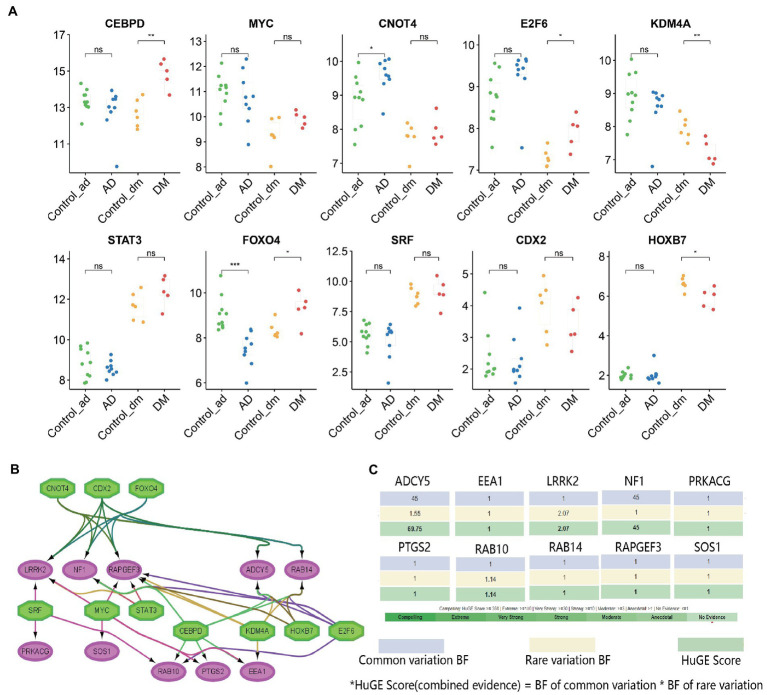
TFs regulatory network and its expression in GSE97760 and GSE95849. **(A)** The expression level of TFs in GSE97760 and GSE95849. The comparison between the two sets of data uses the mean *T*-test. *p < 0.05, **p < 0.01, ***p < 0.001, **(B)** TFs regulatory network. TFs were marked in green, and the hub genes were marked in red, and **(C)** Evidence from human genetics can provide important support for hypotheses about the roles of genes in DM *via* the HuGE Calculator.

Interestingly, after querying the AlzData (high-throughput omics data for AD, http://www.alzdata.org/), we found that *RAPGEF3* was significantly positively correlated with Aβ and Tau, while *NF1* was significantly negatively correlated with Aβ and Tau. Besides, we also use the Attie lab diabetes database (the interactive database of gene expression and diabetes-related clinical phenotypes, http://diabetes.wisc.edu/correl_f2.php) and found that *ADCY5* and *SOS1* had a significant positive association with insulin and body weight, but significantly negatively associated with glucose; On the contrary, *PTGS2*, *EEA1* were significantly and positively associated with glucose, but a significant negative association with insulin and body weight. Human Genetic Evidence Calculator (HuGE Calculator, Evidence from human genetics can provide important support for hypotheses about the roles of genes in disease, https://hugeamp.org/hugecalculator.html?prior), the query results showed *ADCY5*, *NF1* significant associations with type 2 diabetes phenotypes (Figures 9C).

### Key gene validation

Accumulating epidemiological and biochemical evidence suggests that insulin resistance in the brain leads to altered gene expression profiles in the hippocampus and prefrontal cortex of rats, suggesting an association between type 2 diabetes mellitus and Alzheimer’s dementia. The GSE34451 dataset contains triplicates of samples prepared from each brain region of type 2 diabetic Goto-Kakizaki rats and controls animals, providing further experimental evidence for the recently elaborated theory that Alzheimer’s is type 3 diabetes(Abdul-Rahman et al., 2012). Compared with the normal group, *EEA1*, *NF1,* and *RAB14* were significantly up-regulated in the hippocampus. *ADCY5* and *RAPGEF3* were significantly down-regulated in the cortex, *LRRK2*, *RAB10,* and *SOS1* were significantly down-regulated in the striatum, and *NF1* was significantly up-regulated. It has been shown that T2D db/db mice exhibit deficits in short-term and spatial working memory compared to db/m mice. Microarray analysis of hippocampal tissue from T2D db/db mice using the GSE151294 dataset revealed that *EEA1* and *LRRK2* were significantly down-regulated in the hippocampus, while *NF1* was significantly up-regulated.

The above validation results look somewhat different from those of our bioinformatics analysis, which may be due to the different species and subjects tested. In this paper, human blood transcriptomics of DM and AD were studied, and the verification samples were animal brain tissues for verification, but the final results were favorable to us. For example, the mRNA expression trend of *NF1*, *RAB14*, *ADCY5,* and *RAPGEF3* in the blood is the same as that in encephalopathy, suggesting that the purpose of assessing brain state can be achieved by detecting blood-related indicators (Table 2).

**Table 2:** Key gene validation.

Gene	Value of *p*	LogFC	Tissues	Data source
EEA1	0.001	0.91	hippocampus	GSE34451
NF1	0.001	1.05	hippocampus	GSE34451
RAB14	0.015	0.64	hippocampus	GSE34451
ADCY5	0.031	−0.64	prefrontal cortex	GSE34451
RAPGEF3	0.014	−0.76	prefrontal cortex	GSE34451
LRRK2	0.038	−1.14	striatum	GSE34451
NF1	0.030	0.91	striatum	GSE34451
RAB10	0.037	−0.90	striatum	GSE34451
SOS1	0.038	−0.73	striatum	GSE34451
EEA1	0.021	−0.45	hippocampus	GSE151294
LRRK2	0.036	−0.40	hippocampus	GSE151294
NF1	0.020	1.10	hippocampus	GSE151294

### Prediction and verification of TFs

Based on the iRegulon plugin, we found that 10 TFs (NES ≥ 5) may regulate the expression of hub genes (Figure 9A, Supplementary Table S5). Further verification revealed that *CEBPD*, *E2F6*, and *FOXO4* were significantly upregulated in the DM group, while *KDM4A* and *HOXB7* were significantly down-regulated. Similarly, *CNOT4* was significantly upregulated in the AD group, but *FOXO4* was significantly down-regulated. They coordinated in regulating 10 hub genes (Figure 9B).

## Discussion

There is growing evidence that both DM and AD diseases are involved in impaired glucose homeostasis and changes in brain function (Baglietto-Vargas et al., 2016). Current theories and hypotheses suggest that defective insulin signal transduction in the brain causes synaptic dysfunction and cognitive impairment in AD (Pugazhenthi et al., 2017). In addition to common risk factors and clinical symptoms, metabolic defects, such as reduced cerebral glucose metabolism and central insulin resistance, are now considered inherent in AD. Therefore, some researchers believe AD is a “type 3 diabetes” (de la Monte et al., 2018; de la Monte, 2019). There is no doubt that insulin resistance is the bridge between DM and AD. The primary purpose of our study is to identify the common DEGs in AD and DM, reveal potential targets, clarify their common possible pathogenesis, and prevent and treat DM complicated with AD.

In this study, we screened 10 hub genes from 339 overlapping DEGs, including *PTGS2*, *RAB10*, *LRRK2*, *SOS1*, *EEA1*, *NF1*, *RAB14*, *ADCY5*, *RAPGEF3*, and *PRKACG*. GO, and KEGG pathway enrichment disclosed that hub genes were significantly involved in inflammatory, immune, insulin regulation, and hormone metabolism pathways. These 10 genes may play an important role in AD and DM diseases, and play a regulatory role in the occurrence and development of the two diseases.

Endocytosis is an active transport system, which involves the cell membrane of transport molecules entering and leaving the cell through the endocytosis transport system, and all components of the system form the endocytosis pathway (Hansen and Nichols, 2009). When the plasma membrane is partially trapped, endocytosis occurs, in which the contents of the vesicles are internalized through a grid-dependent or grid-independent pathway (Khan and Steeg, 2021). Increasing evidence shows that endocytosis plays a role in Aβ metabolism (Choy et al., 2012). Neurons can clear β-amyloid precursor protein (APP) through endocytosis. Genetic studies have shown that the occurrence and progress of AD are related to some endocytosis-related genes (Hollingworth et al., 2011; Lambert et al., 2013). Therefore endocytosis gene mutations that destroy the physiological function of neurons may contribute significantly to the pathophysiology of AD (Kimura and Yanagisawa, 2018). Recently, a study investigated their association with AD, mild cognitive impairment (MCI) and brain magnetic resonance structural phenotypes by constructing multiple genetic risk scores (MGRS), suggesting that the MGRS capture endocytosis pathway is significantly associated with MCI (Ahmad et al., 2018). However, the effects of endocytosis on multiple genes of brain function seem to be unknown in the AD spectrum (Zhu et al., 2021). It is intriguing that membrane traffic-associated genes, such as RAB10 and EEA1, are included in the 10 hub genes.

Adenosine cyclase type 5 (*ADCY5*) can be used as an effector of neurotransmitters such as the D2 dopamine receptor, mu, δ opioid receptor, and mGluR3 glutamate receptor. It is preferentially expressed in the dorsal striatum and nucleus accumbens, and to a lesser extent in other regions of the brain, such as the prefrontal cortex and cerebellum (Lee et al., 2002; Kim et al., 2017). The change of *ADCY5* expression in the *β*-cells leads to impaired glucose signal transduction, which indicates that *ADCY5* gene polymorphism may affect fasting blood glucose levels and diabetes risk (Ustianowski et al., 2021). For the common influencing factors of AD and DM, such as obesity and depression, the expression of *ADCY5* is increased. *ADCY5* gene expression in adipose tissue is related to obesity in men and mice. In humans and mice, visceral *ADCY5* expression is significantly higher in obese compared to lean individuals, and changes in adipose tissue *ADCY5* expression are related to obesity and fat distribution, but not to impaired glucose metabolism and T2DM (Knigge et al., 2015).

LncRNA *PTGS2* can damage islet *β*-cell function by regulating miR-146a-5p and upregulating RBP4, suggesting that LncRNA *PTGS2* has potential value in the diagnosis of DM (Chen et al., 2021). Elevated levels of cyclooxygenase-2 (*COX*-*2*/*PTGS2*) and prostaglandins (*PGs*) are involved in the pathogenesis of AD, *COX-2* dysregulation influences abnormal cleavage of the *β*-amyloid precursor protein, aggregation, and deposition of *β*-amyloid plaques and the inclusion of phosphorylated tau in neurofibrillary tangles. The mechanisms of *PTGS2* regulation of AD may include neuroinflammation, oxidative stress, synaptic plasticity, neurotoxicity, autophagy, and apoptosis (Guan and Wang, 2019).

*RAB10* is a small Rab GTP enzyme involved in vesicle transport and has recently been identified as a new protein related to AD. Interestingly, *RAB10* is the key substrate of leucine-rich repetitive kinase 2 (*LRRK2*) (Healy et al., 2008). Besides, *RAB10* phosphorylation leads to neurodegeneration, which may be responsible for vesicular transport aberration observed in AD (Tavana et al., 2019).

Early endosomal antigen 1 (*EEA1*) was significantly increased in the human cerebrospinal fluid from AD patients compared with neurological controls, and *EEA1* levels correspond to the increased total-tau levels (Armstrong et al., 2014). *EEA1* gene is a candidate mutation for susceptibility to diabetes in the Japanese population, which has been confirmed by a genetic background of familial clustering of diabetes using genome-wide linkage analysis combined with exome sequencing (Tanaka et al., 2013). For DM patients, *SOS1* was statistically significantly associated with gestational diabetes mellitus risk at the gene level (Chen et al., 2018).

**Figure 10:**
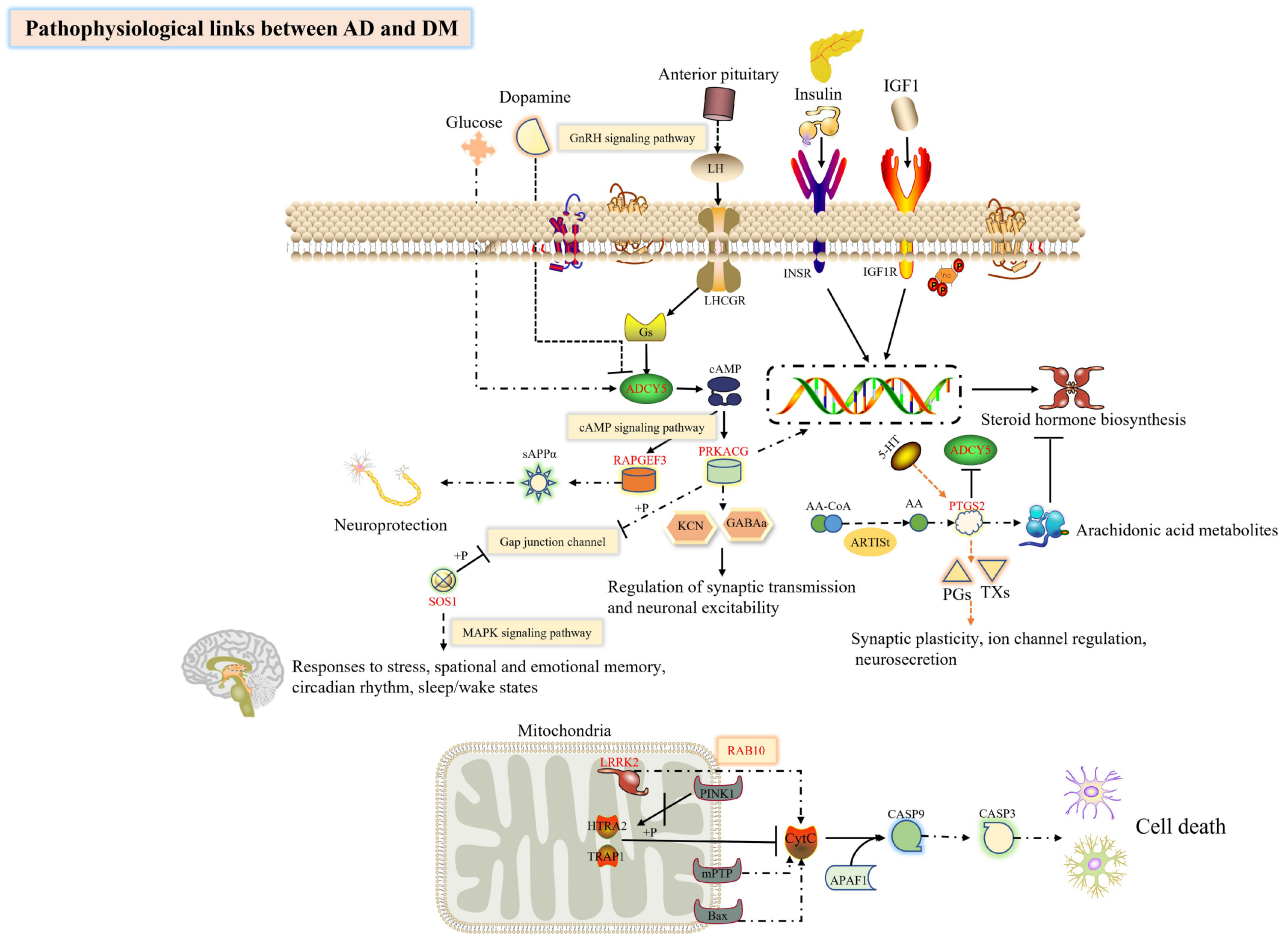
Exploring the hub genes related to the pathogenesis of DM complicated with AD by bioinformatics analysis. This figure was drawn by the ScineceSlides plugin.

Based on the above literature, we speculate (Figure 10) that *ADCY5* stimulates *RAPGEF3* and *sAPPα* through the cAMP signal pathway to play a neuroprotective effect, while *PTGS2* can inhibit the activity of *ADCY5*. The trend of the expression of these genes is consistent with our conjecture, so we have reason to suggest that the loss of control of these genes leads to the destruction of neuroprotection; Both *PRKACG* and *SOS1* can inhibit the Gap junction channel, destroying the blood–brain barrier and brain disease; *LRRK2* and substrate *RAB10* jointly mediate apoptosis, but this study found that the expression of these two genes in DM and AD were significantly increased, suggesting that their elevation led to neuronal apoptosis and promoted the transformation of DM to AD.

In addition, according to the DEGs of AD and DM, the small molecule drug prediction was carried out through the CMap database, and the small molecule compound, such as L-690488, exemestane, and BMS-345541 may reverse the pathology of AD and DM, was identified. Compared with other bioinformatics studies, our research focus is slightly different. In addition to exploring the common hub genes and biological pathways involved in AD and DM, we have also explored possible therapeutic drugs and related TFs. By building complex interactive networks, it is easy to get their common DEGs and identify potential key targets. This comprehensive bioinformatics approach is reliable and informative in a variety of diseases (Su et al., 2021; Ye et al., 2021). In addition, we also analyzed the related TFs expression levels in the original data set, to explore whether TFs will also be affected by the disease. Although previous studies have explored the central genes related to AD and DM respectively, this study focuses on the molecular mechanism of DM complicated with AD, providing a potential direction for the mechanism of diseases with complications (Gudala et al., 2013; Li et al., 2020; Zhu et al., 2020).

However, we have to point out some limitations of this study. First of all, this is a microarray data analysis study, which theoretically belongs to the retrospective study, although this approach can speed up our work efficiency in discovering disease mechanisms, more external verification is needed to verify the key objects of our findings; Secondly, the development of DM into AD is a dynamic and slow process. In the future, we will study the gene matrix of DM complicated with AD, and look for the marker genes of this process (perhaps the key genes *ADCY5*, *PTGS2*, *RAB10*, etc. that we have studied play a role in this dynamic process), thereby effectively controlling the development of DM symptoms; Thirdly, the function of hub genes in disease needs further verification by corresponding biological models. Whether these genes have positive significance for clinical evaluation remains to be explored, which will be a challenge for our future work in AD and DM.

## Conclusion

In conclusion, we explored the possible DEGs of AD and DM, and performed routine bioinformatics analysis and PPI network construction. As we expected, AD and DM contribute various commonplace pathogenic mechanisms, which perchance voluntary by individual hub genes. Glucose homeostasis and changes in brain function, *NF1*, *RAB14*, *ADCY5,* and *RAPGEF3* could be the focus of later studies (Figure 10). Up to now, the connection between essential genes and immune infiltration of AD and DM has been infrequently reported. Whether the key genes have clinical diagnostic significance and whether the factors related to immune infiltration are conducive to the diagnosis of AD or DM remains to be explored. This study states a new concept for the continued exploration of the molecular mechanism of DM accompanied by AD or other diseases.

## Data availability statement

The datasets presented in this study can be found in online repositories. The names of the repository/repositories and accession number(s) can be found in the article/Supplementary material.

## Author contributions

X-fX and X-rL developed a major research plan. W-jX and XW analyzed the data. S-qC, Y-yB, and C-sL drew charts. M-nL and X-wY wrote manuscripts. L-lY and X-xW helped to collect data and references. JW and S-yZ implemented corrections in the manuscript. All authors contributed to the article and approved the submitted version.

## Funding

This study was supported by the National Natural Science Foundation of China (no. 81973480) and the National Key Research and Development Program of China (no. 2019YFC1711500).

## Conflict of interest

The authors declare that the research was conducted in the absence of any commercial or financial relationships that could be construed as a potential conflict of interest.

## Publisher’s note

All claims expressed in this article are solely those of the authors and do not necessarily represent those of their affiliated organizations, or those of the publisher, the editors and the reviewers. Any product that may be evaluated in this article, or claim that may be made by its manufacturer, is not guaranteed or endorsed by the publisher.

## Footnotes

^1^ http://www.ncbi.nlm.nih.gov/geo

^2^ https://www.omicshare.com

^3^ https://www.chiplot.online

^4^ http://www.cytoscape.org

^5^ http://www.genemania.org/

^6^ https://go.drugbank.com/

## Abbreviations

AD: Alzheimer’s disease
DM, Type 2 Diabetes Mellitus
GO, Gene Ontology
KEGG, Kyoto Encyclopedia of Genes and Genomes Pathway
PPI, Protein–protein Interaction
Aβ, Amyloid beta
DEGs, Differentially expressed genes
GEO, Gene Expression Omnibus
TFs, transcription factors
STRING, Search Tool for the Retrieval of Interacting Genes
CMap, Connectivity Map
ImmuCellAI: Immune Cell Abundance Identifier
